# Early oral intake and early removal of nasogastric tube post‐esophagectomy: A systematic review and meta‐analysis

**DOI:** 10.1002/cnr2.1538

**Published:** 2021-09-07

**Authors:** Suha Kaaki, Emma J. M. Grigor, Donna E. Maziak, Andrew J. E. Seely

**Affiliations:** ^1^ Department of Surgery, Division of Thoracic Surgery, The Ottawa Hospital Ottawa Canada; ^2^ Faculty of Medicine University of Ottawa Ottawa Canada; ^3^ Clinical Epidemiology Program Ottawa Hospital Research Institute Ottawa Canada

**Keywords:** anastomotic leak, aspiration pneumonia, early oral intake, esophagectomy, nasogastric tube, perioperative complications

## Abstract

**Background:**

Early oral intake (EOI: initiated within 1 day) and early nasogastric tube removal (ENR: removed ≤2 days) post‐esophagectomy is controversial and subject to significant variation.

**Aim:**

Our aim is to provide the most up‐to‐date evidence from published randomized controlled trials (RCTs) addressing both topics.

**Methods:**

We searched MEDLINE and Embase (1946‐06/2019) for RCTs that investigated the effect of EOI and/or ENR post‐esophagectomy with gastric conduit for reconstruction. Our main outcomes of interest were anastomotic leak, aspiration pneumonia, mortality, and length of hospital stay (LOS). Pooled mean differences (MD) and risk ratios (RR) estimates were obtained using a DerSimonian random effects model.

**Results:**

Two reviewers screened 613 abstracts and identified 6 RCTs eligible for inclusion; 2 regarding EOI and 4 for ENR. For EOI (2 studies, *n* = 389), was not associated with differences in risk of: anastomotic leak (RR: 1.01; 95% CI: 0.407, 2.500; I^2^: 0%), aspiration pneumonia (RR: 1.018; 95% CI: 0.407, 2.500), mortality (RR: 1.00; 95% CI: 0.020, 50.0). The LOS was significantly shorter in the EOI group: LOS (MD: −2.509; 95% CI: −3.489, −1.529; I^2^: 90.44%). For ENR (4 studies, *n* = 295), ENR (removed at POD0‐2 vs. 5–8 days) was not associated with differences in risk of: anastomotic leak (RR: 1.11; 95% CI 0.336, 3.697; I^2^: 25.75%) and pneumonia group (RR: 1.11; 95% CI: 0.336, 3.697; I^2^: 25.75%), mortality (RR: 0.87; 95% CI: 0.328, 2.308; I^2^: 0%)or LOS (MD: 1.618; 95% CI: −1.447, 4.683; I^2^: 73.03%).

**Conclusions:**

Our analysis showed that EOI as well as ENR post‐esophagectomy do not significantly increase the risk of anastomotic leak, pneumonia, and mortality. The LOS was significantly shorter in the EOI group, and there was no significant difference in the ENR group. A paucity of RCTs has evaluated this question, highlighting the need for further high‐quality evidence to address these vital aspects to post‐esophagectomy care.

**Systematic review registration:**

CRD42019138600

## 1 BACKGROUND

Esophageal cancer is among the fastest growing cancers and is associated with a high fatality rate. Currently, esophagectomy is the primary treatment for resectable esophageal adenocarcinoma, following neoadjuvant chemotherapy and/or radiotherapy in most patients. Esophagectomy is a morbid procedure and may be associated with a long hospital stay and slow recovery. Patients may be malnourished secondary to the morbidity of their presenting illness and possible neoadjuvant therapies. Moreover, esophagectomy changes the anatomy of the upper gastro‐intestinal tract forever. Post‐esophagectomy optimal nutrition is essential for healing and for overall advancement of the patients' post‐operative course. Hence, addressing the optimal care after this morbid surgery is an important topic.

There are multiple controversies regarding the best practice of care post‐esophagectomy, two of which we focus on in this paper, namely early oral intake (EOI), defined as initiating oral intake of clear fluids on post‐operative (post‐op) day 1, and early nasogastric tube removal (ENR), defined as removal of the nasogastric tube by post‐op day 2. Traditionally, oral intake is delayed until a test of the anastomosis has been performed. Indeed, some studies suggested that the early advancement of oral intake might predispose to anastomotic leaks and aspiration pneumonia.^1^, ^2^ Conversely, other studies utilizing an enhanced recovery after surgery (ERAS) post‐esophagectomy framework, showed that the initiation of EOI was safe and decreased the length of hospital stay.^3^ Nasogastric tubes are inserted post‐esophagectomy to decompress air and fluid within the gastric conduit which is thought to help prevent anastomotic leak and aspiration of gastric contents.^4^ However, these tubes can cause considerable amount of discomfort to the patients, and can induce vomiting which contribute to the development of aspiration pneumonia.^5^ There are strong opinions, yet no consensus regarding these two controversies regarding post‐esophagectomy care. Thus, for the first time, our aim is to present provide a comprehensive summary and meta‐analysis of all published randomized controlled trials (RCTs) that aimed to address two important aspects of post‐esophagectomy care, which are the safety of EOI and the need for a nasogastric tube decompression post operatively.

## 2 METHODS

This systematic review and meta‐analysis were conducted according to the preferred reporting items for systematic reviews and meta‐analyses guidelines (PRISMA checklist)^6^ (Supporting information Appendix 1). The protocol is available in the International Prospective Register of Systematic Reviews (CRD42019138600).

### 2.1 Search strategy

MEDLINE (OVID interface, including in‐process and Epub ahead of print) and Embase (OVID interface) databases were searched from 1946 to February 2019 (Supporting information Appendix 2). The literature search results were uploaded and reviewed using Covidence Software (Covidence, Melbourne, Australia).

### 2.2 Selection criteria

Search results and full‐text articles meeting full eligibility criteria were reviewed independently and in duplicate. Potentially relevant studies were screened by title and abstract (stage 1) followed by full‐text article screening to assess full eligibility (stage 2). Two review authors assessed the eligibility of full reports (S. K., E. G.). Any disagreement was resolved through discussion with a third reviewer (A. S.). The reasons for excluding studies were recorded. RCTs that evaluated the effects of ENR or EOI intake following esophagectomy were included. Properly conducted RCTs are the gold standard for evaluating the effects of an intervention.^7^ Review articles, editorials, preclinical studies, observational studies, and abstracts were excluded.

### 2.3 Outcome justification and prioritization

The primary outcomes of interest were anastomotic leak and aspiration pneumonia. Anastomotic leak was defined as the presence of extraluminal collections of air or contrast, excess bile‐stained fluid on drainage, or a combination. The secondary outcomes of interest were mortality length of hospital stay, and other post‐operative complications.

### 2.4 Data extraction

Standardized forms were created to inform data extraction from the eligible studies. Patient characteristics and demographics, study methodology, intervention characteristics, and outcomes of interest were recorded. The study and patient characteristics for the included studies were also recorded. This included country of origin, number of patients studied (intervention and/or control arm), the start time of oral intake, type of oral intake, timing of nasogastric tube removal, and postoperative complications. Disagreements were resolved through discussion with a third‐party member.

### 2.5 Summary measures and synthesis of results

DerSimonian and Laird's random‐effects method was used to pool relative risk effect estimates with corresponding 95% CIs for dichotomous variables.^8^ Continuous measures were reported for individual studies as a mean with ±SD or a median with interquartile range (IQR) or the overall range from minimum to maximum. The pooled mean difference between the length of stay in the intervention and control groups was determined using a DerSimonian and Laird's random‐continuous effects method. Studies that reported median with IQR were excluded from the pooled mean difference estimation for the length of stay. The heterogeneity of effect sizes for pooled estimates was assessed using the Cochrane I^2^ statistic. The following thresholds were used to describe the I^2^ threshold: 0%–40% (low heterogeneity), 30%–60% (moderate heterogeneity), 50%–90% (substantial heterogeneity), and 75%–100% (considerable heterogeneity). Open Meta‐Analyst was used to generate forest plots, heterogeneity, and effect estimates for risk ratios and mean differences (Open‐source, USA).^9^

### 2.6 Risk of bias

The Cochrane revised risk of bias tool for randomized trials was used to evaluate the individual risk of bias for RCT studies reviewed.^10^ Within each risk of bias domain, a series of questions (“signaling questions”) were chosen to elicit information about features of the trial that were felt to be relevant to the risk of bias. Publication bias was included in the assessment. Judgment is classified as “low,” “high,” or as having “some concerns.” Meta‐bias (or risk of bias across studies) was summarized by pooling the individual study risk of bias for each risk of bias domain.

### 2.7 Grading of recommendations, assessment, development, and evaluations

The quality of the treatment effects was graded by using a systematic and comprehensive approach known as GRADE.^11^ GRADE provides a reproducible and transparent framework for grading the quality of evidence or certainty in the evidence. The quality of evidence reflects the extent to which we are confident that an estimate of the effect is correct. High grade of evidence means the true estimate lies close to the estimate of effect; moderate grade means that the true effect is likely to be close to the estimate of the effect; low grade means that the effect estimate may substantially differ from the true estimate of the effect; very low grade means we have little confidence in the effect estimate.^11^

## 3 RESULTS

**FIGURE 1:**
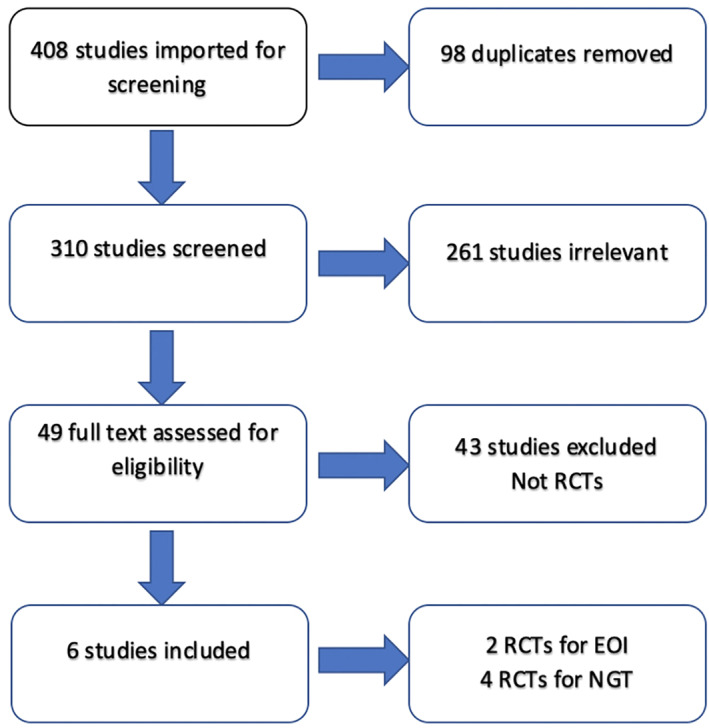
Preferred reporting items for systematic reviews and meta‐analyses guidelines (PRISMA)

The systematic searches returned a total of 408 citations. Following deduplication, abstracts were reviewed, and 310 full manuscripts were identified as potentially eligible. Six RCT studies met eligibility for inclusion (*n* = 684 patients). All six studies were included in our meta‐analysis, as shown in the PRISMA flow diagram (Figure 1).

Studies were published between 1996 and 2019, with sample sizes ranging from 34 to 280 participants. The mean age of participants was similar across studies, ranging from 53.4 to 66.4 years. Studies were performed in Iran (2 studies, 33%), China (1 study, 17%), India (1 study, 17%), United Kingdom (1 study, 17%), and Japan (1 study, 17%). The number of male participants was higher than female participants in all six studies. Follow‐up was only reported in one study at 24 weeks. The incidence of anastomotic leak ranged from 3.9% to 15%. The patient characteristics of the included studies are provided in Table 1.

**TABLE 1:** Study characteristics for EOI and ENR

First author, year	Country	Total (*N*)	Intervention group (*n*)	Age, mean years (±SD)	Male/female ratio (*n*/*n*)	Follow‐up, mean weeks (±SD)
EOI						
Mahmoodzadeh 2015	Iran	109	54	C: 66.4 ± 7.7 I: 64.2 ± 8.2	C: 29/26 I: 29/25	NR
Sun 2018	China	280	140	C: 63 I: 63	C: 103/37 I: 92/48	24 weeks
ENR						
Daryaei 2009	Iran	40	22	C: 58.4 ± 10.3 I: 60.1 ± 8.1	NR	NR
Hayashi 2019	Japan	71	37	T: 63.04 ± 7.8 C: 62.47 ± 7.2 I: 63.57 ± 8.4	T: 62/9 C: 30/7 I: 32/2	NR
Mistry 2012	India	150	75	C: 56.7 I: 53.4	C: 51/24 I: 51/24	NR
Shackcloth 2006	United Kingdom	34	22	C: 61 I: 62	C: 18/6 I: 9/3	NR

Abbreviations: C, control; ENR, early nasogastric tube removal; EOI, early oral intake; I, intervention; NR, not reported; T, total.

Of six studies reviewed, 2 (33%) studies investigated EOI (*n* = 389 patients) and 4 (67%) studies investigated ENR (*n* = 295 patients). Three studies (50%) used hand sewn anastomosis only, one study (17%) used both hand sewn and stapled anastomosis, one study (17%) used stapled anastomosis only, and one study (17%) did not report the type of anastomosis used. Four studies (67%) performed cervical anastomosis, and two studies (33%) a thoracic anastomosis. Among the EOI studies, clear fluids were administered to all patients on POD1. Overall, 121 (range 12–37) patients had neoadjuvant chemotherapy and 5 (0–2) patients had neoadjuvant chemoradiotherapy in the intervention groups; 127 (range 9–47) patients had neoadjuvant chemotherapy and 5 (0–2) patients had neoadjuvant chemoradiotherapy in the control groups. Among the EOI studies, 15 patients (3.8%, 2 studies) had their nasogastric tube reinserted in the intervention group compared to 36 patients (9.2%, 2 studies) in the control group; 18 patients (4.6%, 2 studies) had repeat nil per os in the intervention group and 17 patients (16%, 1 study) in the control group. The length of stay ranged from 6 to 25.7 days. The study intervention characteristics are outlined in Table 2.

**TABLE 2:** Intervention characteristics for EOI and ENR

First author, year	First PO (POD)	First PO type	NGT re‐inserted (*n*)	Repeat NPO (*n*)	Anastomotic location	Anastomosis type	Neoadjuvant chemotherapy (*n*)	Neoadjuvant chemoradiotherapy (*n*)	Length of stay, days
EOI									
Mahmoodzadeh 2015	1	Clear fluid	C: 25 I: 7	C: 17 I: 8	Thoracic	NR	C: 14 I: 12	C: 0 I: 0	C: 8 I: 6
Sun 2018	1	Clear fluid	C: 11 I: 8	C: NR I: 10	Cervical	Hand sewn	C: 47 I: 36	C: 2 I: 2	C: 10 I: 7

First author, year	NGT insertion (Yes, No)	NGT removal Mean days (± SD)	Feeding with NGT in place (Yes, No)	NGT Re‐inserted (*n*)	Anastomotic location	Anastomosis type	Neoadjuvant chemotherapy (*n*)	Neoadjuvant chemoradiotherapy (*n*)	Length of stay, mean (±SD) or median (IQR)
ENR									
Daryaei 2009^a^	Yes	C: 4.2 ± 1.3 I: 0	No	C: 0 I: 1	Cervical	Hand sewn	NR	NR	C: 10.9 (± 3.5) I: 13.9 (± 8.2)
Hayashi 2019^b^	Yes	C: 7 I: 1	No	C: 1 I: 1	Cervical	Hand sewn	C: 19 I: 22	C: 2 I: 2	C: 29.4 (±18.06) I: 25.7 (±12.76)
Mistry 2012	Yes	C: 6–10 I: 2	No	C: 7 I: 23	Cervical	Hand sewn, stapled	C: 37 I: 35	C: 1 I: 1	C: 12 (10–17) I: 12 (9–17)

Abbreviations: C, control; ENR, early nasogastric tube removal; EOI, early oral intake; I, intervention; NGT, nasogastric tube; NPO, nil per os; NR, not reported; PO, oral; POD, post‐operative day.

^a^ Reported median (IQR).

^b^ Reported mean (±SD).

### 3.1 Primary outcome

#### 3.1.1 Anastomotic leak

**FIGURE 2:**
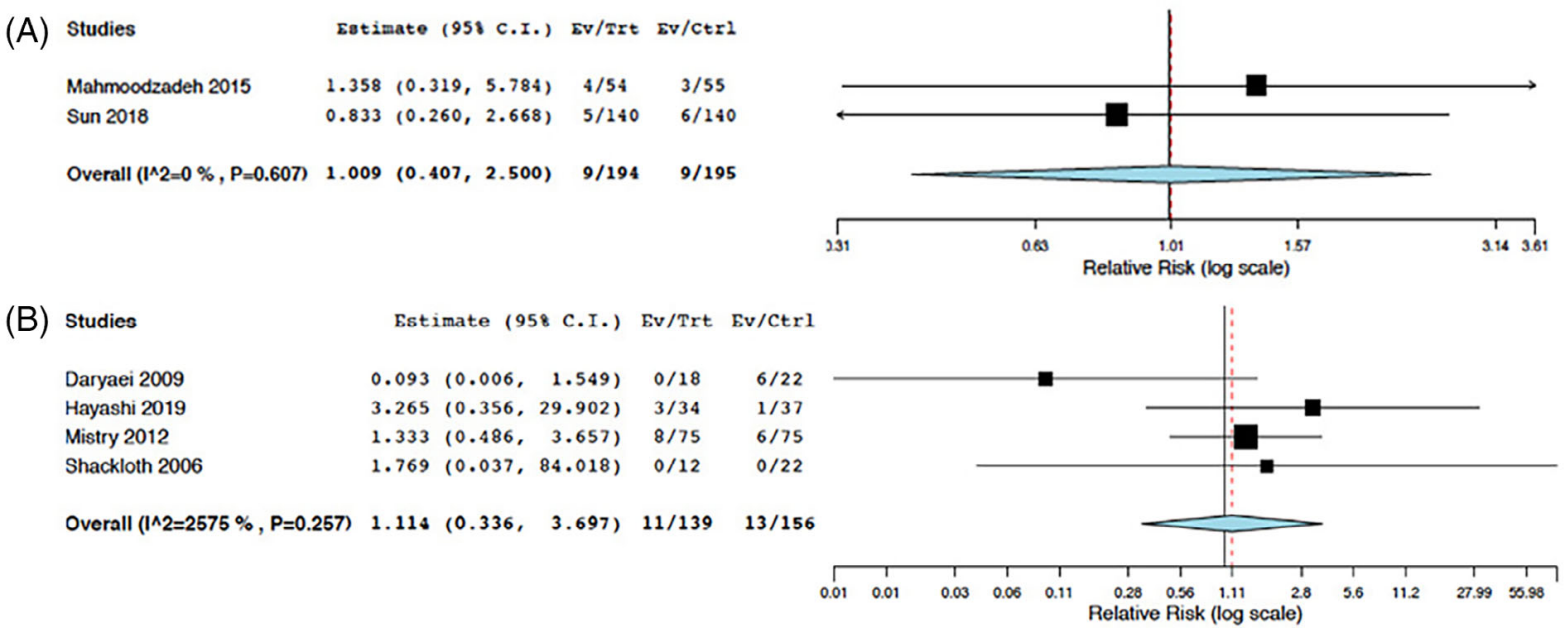
Pooled risk ratio for anastomotic leakage according to intervention type (six meta‐analyzed studies). Intervention compared to control groups for early nasogastric tube removal (ENR) (A) and early oral intake (EOI) (B)

The results comparing the difference in proportion of anastomotic leak events among EOI and ENR are provided in Table 3. In the EOI studies, 18 patients (4.6%) had anastomotic leak (*n* = 389 patients total); there was no statistically significant difference comparing intervention and control groups (*p* > .05). In the ENR studies, 39 patients (12%) had anastomotic leak (*n* = 332 patients total); there was no statistically significant difference comparing intervention and control groups (*p* > .05). Pooled risk ratio (RR) estimates for 3 ENR and 2 EOI studies were obtained for anastomotic leak as shown in Figure 2. Patients that had ENR had a similar risk of anastomotic leak compared to the control group (RR: 1.11; 95% CI: 0.336, 3.697; I^2^: 25.75%). Patients that had EOI had a similar risk of anastomotic leak compared to the control group (RR: 1.01; 95% CI: 0.407, 2.500; I^2^: 0%).

**TABLE 3:** Event rates for primary outcomes

First author, year	Total patients (*N*)	Total events *n* (%)	Anastomotic leak					Total events n (%)	Aspiration pneumonia				
			Intervention total (*n*)	Events (%)	Control total (*n*)	Events (%)	*p* value		Intervention total (*n*)	Events (%)	Control total (*n*)	Events (%)	*p* value
EOI													
Mahmoodzadeh 2015	109	7 (6.4)	54	4 (7.4)	55	3 (5.4)	.671	0 (0)	54	0 (0)	55	0 (0)	1.00
Sun 2018	280	11 (3.9)	140	5 (3.6)	140	6 (4.3)	.764	10 (7.1)	140	10 (7.1)	NR	NR	N/A
*Overall*	389	18 (4.6)	194	9 (4.6)	195	9 (4.6)	1.00	10 (2.6)	194	10 (5.2)	55	0 (0)	.085
ENR													
Daryaei 2009	40	6 (15)	18	0 (0)	22	6 (27)	.018*	2 (5.0)	18	2 (11)	22	0 (0)	.115
Hayashi 2019	71	4 (5.6)	34	3 (8.8)	37	1 (2.7)	.268	15 (21.1)	34	7 (21)	37	8 (22)	.234
Mistry 2012	150	14 (9.3)	75	8 (11)	75	6 (8.0)	.838	43 (29)	75	18 (24)	75	25 (33)	.224
Shackcloth 2006	34	0 (0)	12	0 (0)	22	0 (0)	.532	11 (32)	12	7 (58)	22	4 (18)	.019*
*Overall*	332	39 (12)	139	11 (7.9)	156	13 (8.3)	.826	45 (14)	139	34 (24)	156	37 (24)	1.00

Abbreviations: C, control; ENR, early nasogastric tube removal; EOI, early oral intake; I, intervention; NGT, nasogastric tube; NR, not reported; TC, total number of patients in control group; TI: total number of patients in intervention group.

^*^ Chi‐square test was performed to obtain *p* values (significant <.05).

#### 3.1.2 Aspiration pneumonia

**FIGURE 3:**
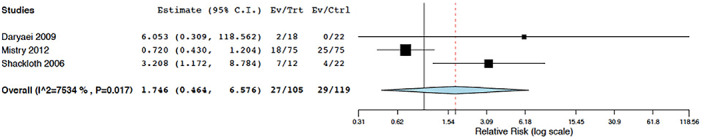
Pooled risk ratio for aspiration pneumonia (six meta‐analyzed studies). Intervention compared to control groups for early nasogastric tube removal (ENR)

The results comparing the difference in proportion of aspiration pneumonia events among EOI and ENR are provided in Table 3. In the EOI studies, 10 patients (2.6%) had aspiration pneumonia (*n* = 389 patients total); There was no statistically significant difference comparing intervention and control groups (*p* > .05). In the nasogastric intubation studies, 45 patients (14%) had aspiration pneumonia (*n* = 332 patients total); There was no statistically significant difference comparing intervention and control groups (*p* > .05). Pooled risk ratio (RR) estimate for 3 ENR studies was obtained for aspiration pneumonia as shown in Figure 3. Patients that had ENR had a similar risk of aspiration pneumonia compared to the control group (RR: 1.11; 95% CI: 0.336, 3.697; I^2^: 25.75%). Sun et al. were the only EOI study that reported aspiration pneumonia, and the risk ratio showed a similar risk of aspiration pneumonia compared to the control group (RR: 1.018; 95% CI: 0.407, 2.500).

#### 3.1.3 Secondary outcomes

**FIGURE 4:**
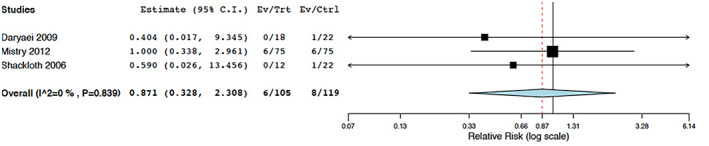
Pooled risk ratio for mortality (six meta‐analyzed studies). Intervention compared to control groups for early nasogastric tube removal (ENR) (A) and early oral intake (EOI) (B)

**FIGURE 5:**
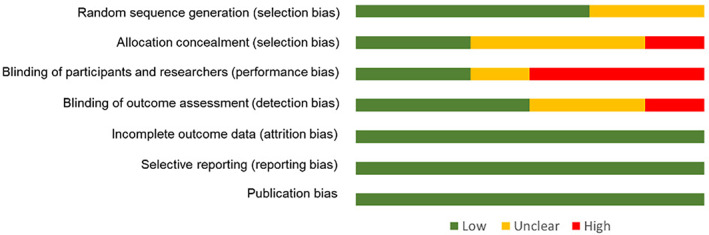
Pooled mean difference for length of stay (six meta‐analyzed studies). Intervention compared to control groups for early nasogastric tube removal (ENR) (A) and early oral intake (EOI) (B)

Pooled risk ratio (RR) estimate for 3 ENR was obtained for mortality, shown in Figure 4. Patients that had ENR had a similar mortality to the control group (RR: 0.871; 95% CI: 0.328, 2.308; I^2^: 0%). Mahmoodzadeh et al. 2015 were the only EOI study reported mortality, patients that had EOI had a similar risk of mortality compared to the control group (RR: 1.00; 95% CI: 0.020, 50.0). Pooled mean difference (MD) estimates for 2 ENR was obtained for length of stay as shown in Figure 5. Patients that had ENR had a similar length of stay compared to the control group following esophagectomy (MD: 1.618; 95%CI: −1.447, 4.683; I^2^: 73.03%). Patients who had EOI had a significantly decreased LOS compared to the control group (MD: −2.509; 95% CI: −3.489, −1.529; I^2^: 90.44%). Both groups were comparable with regards to other complications.

#### 3.1.4 Risk of bias

Overall, the meta‐bias analysis revealed that there was low risk of bias for incomplete outcome reporting, selective reporting, publication bias, and random sequence generation. Allocation concealment had unclear risk of bias due to two (33%) studies not reporting whether blinding of outcome assessment was performed, and one (17%) study reported that no blinding was use. Blinding of study participants showed unclear risk of bias due to two (33%) studies not reporting whether blinding of outcome assessors was performed and one (17%) reporting that there was no blinding. There was high risk of bias of outcome assessment due to three (50%) studies reporting no blinding of outcome assessors and one (17%) not reporting whether blinding was performed. The risk of bias results is summarized in Figure 5 (individual study risk of bias summarized in Supporting information Appendix 3).

## 4 GRADE

The GRADE results for anastomotic leak were grouped according to intervention type. Overall, there was “low” quality of evidence for the pooled anastomotic leak results in the ENR group; this was explained by the unclear risk of bias and the imprecision in the effect estimate for anastomotic leak. The unclear risk of bias in ENR studies decreased the quality by one level; this was due to the lack of allocation concealment and unclear/high risk of bias for blinding of study participants and investigators. The high imprecision in the ENR studies reduced the quality by one level; this was due to the fact that the 95% confidence interval was not statistically significant. Overall, there was “very low” quality of evidence for the anastomotic leak results pooled in the EOI studies; the high risk of bias in EOI studies decreased the quality by two levels; this was due to the lack of allocation concealment and unclear/high risk of bias for blinding of study participants and investigators. The high imprecision in the ENR studies reduced the quality by one level; this was due to the fact that the 95% confidence interval was not statistically significant. The heterogeneity was moderate in the EOI studies, which decreased the quality by one more level. The summary for the GRADE results is provided in Supporting information Appendix 4.

## 5 DISCUSSION

Despite multiple previous studies that showed that starting clear fluids on post‐op day 1 and removal of the nasogastric tube by post‐op day 2 or even not inserting a nasogastric tube post‐esophagectomy is a safe practice, many surgeons are reluctant to adopt such practice for the fear of anastomotic leak and aspiration pneumonia. As these complications have deleterious effects on compromised patients recovering post‐esophagectomy, our meta‐analysis focused on these two adverse events. We found similar risk of anastomotic leak, aspiration pneumonia, and mortality comparing patients who had ENR or EOI to the control group. Our results also showed that the length of hospital stay was significantly shorter in the group of patients who had EOI post‐operatively. There was low quality of evidence to support the similar risk of anastomotic leak in patients that had ENR compared to the control group. There was very low quality of evidence to support the similar risk of anastomotic leak in patients that had EOI compared to the control group. Overall, the practice of EOI and ENR was not inferior to the usual practice based on these data and appears to offer benefit to decreasing length of stay.

These findings are in keeping with ERAS protocols, which have been demonstrated to be associated with a decreased rate of complications and decreased length of hospital stay in other gastrointestinal surgeries like colorectal surgery.^12^ The evidence of implementation of ERAS protocol post‐esophagectomy is scarce, but multiple reviews showed that EOI was associated with a decrease in the length of hospital stay. Blom et al., showed in their study that the ERAS protocol and, specifically, EOI on POD5 post‐esophagectomy resulted in small but significant reduction in overall hospital stay.^3^ Another study by Cao et al., stated that EOI, early removal of chest tubes and early ambulation as part of the ERAS protocol facilitated early discharge of patients.^13^ In these two studies, ENR^3^ or no insertion of one post operatively^13^ was part of the ERAS protocol. In a study done by Sun et al., EOI on POD1 was associated with improved bowel function recovery, decreased length of hospital stay, and was not associated with increased in the incidence of post‐operative complications.^14^ Our meta‐analysis suggests comparable safety among patients that receive ENR or EOI, and indeed possible benefit compared to the standard of care control groups.

This practice provides additional evidence to the ERAS protocol that is being implemented more day by day. ENR spares the patient the significant discomfort and the rare complications caused by its presence and insertion. In addition, ENR may help the swallowing mechanism to normalize. EOI can contribute to normalizing the swallowing mechanism as well, help with oral hygiene, help avoid pharyngeal muscle atrophy, and does not appear to affect the gastroesophageal anastomosis as previously stated. Overall, the lack of significant findings seems to suggest that there is no significant difference in the risk of anastomotic leak for patients that have ENR or EOI (compared to the control). However, our findings should be interpreted with caution due to the very low to low quality of evidence in the pooled effect estimates.

Our study addresses a controversial topic in post‐esophagectomy care. It combines the results of six RCTs. To our knowledge, no other meta‐analysis addresses these two integrated aspects of post‐operative care after this morbid surgery. However, this study was limited by the low number of studies available in the literature and the small sample sizes of patients included in each study. In addition, none of the studies in this analysis were conducted in North America, which means that findings may not necessarily be representative of North American populations undergoing the treatment. Caution is required when generalizing these results to the North American population. Last, while EOI and ENR may be beneficial for patients with low risk of anastomotic leak, there may be patients where there are concerns regarding this adverse event (e.g., due to impaired conduit blood supply) which might suggest avoiding this approach; however this requires further investigation. Regardless, we note the literature addressing these two important aspects of post‐esophagectomy care is sparse, and larger studies are warranted to help better understand current controversies surrounding these practices. We note that the NUTRIENT I and NUTRIENT II trials are ongoing to help clarify the best route of feeds post‐esophagectomy.^15^, ^16^

## 6 CONCLUSION

Our systemic review and meta‐analysis summarized the results of available RCTs addressing the safety of EOI and ENR post‐esophagectomy. These early interventions did not increase the risk of anastomotic leak, aspiration pneumonia, or perioperative mortality. EOI appears to be associated with decreased hospital stay. Since the findings showed low quality evidence, further research is recommended. Quality of evidence profiles presented in our review may help inform future guideline recommendations surrounding the safety of EOI and the need for nasogastric tube decompression post‐esophagectomy. Due to the “very low” quality of evidence that EOI did not increase the risk of anastomotic leak, we cannot make our conclusions with a high level of certainty.

## CONFLICT OF INTEREST

The authors have stated explicitly that there are no conflicts of interest in connection with this article.

## AUTHOR CONTRIBUTIONS

**Emma Grigor:** Conceptualization (supporting); data curation (supporting); formal analysis (lead); methodology (equal). **Donna Maziak:** Conceptualization (lead); data curation (supporting); formal analysis (supporting); methodology (lead); project administration (lead); supervision (lead); validation (lead). **Andrew Seely:** Conceptualization (lead); data curation (supporting); formal analysis (supporting); methodology (lead); project administration (lead); supervision (lead); validation (lead).

## ETHICAL STATEMENT

Not applicable.

## Supporting information


**Appendix 1.** PRISMA Checklist
**Appendix 2.** Literature search strategy
**Appendix 3.** A revised tool to assess risk of bias in randomized trials (RoB 2)^1^
**Appendix 4.** Grading of Recommendations, Assessment, Development and Evaluations for Anastomotic Leak

## Data Availability

The dataset generated and analyzed in our review are available from the corresponding author on reasonable request.
