# Supplementary material for: Early oral intake and early removal of nasogastric tube post‐esophagectomy: A systematic review and meta‐analysis
Source: Cancer Rep (Hoboken). 2021 Sep 7;5(5):e1538. doi: 10.1002/cnr2.1538 (PMC9124520; doi:10.1002/cnr2.1538)
Supplement: Supplementary file 1 — Appendix 1. PRISMA Checklist Appendix 2. Literature search strategy Appendix 3. A revised tool to assess risk of bias in randomized trials (RoB 2)1 Appendix 4. Grading of Recommendations, Assessment, Development and Evaluations for Anastomotic Leak [file CNR2-5-e1538-s001.docx]

**Appendix 1. PRISMA Checklist**

| **Section/topic** | **#** | **Checklist item** | **Reported on page #** |
| --- | --- | --- | --- |
| **TITLE** | | |  |
| Title | 1 | Identify the report as a systematic review, meta-analysis, or both. | 1 |
| **ABSTRACT** | | |  |
| Structured summary | 2 | Provide a structured summary including, as applicable: background; objectives; data sources; study eligibility criteria, participants, and interventions; study appraisal and synthesis methods; results; limitations; conclusions and implications of key findings; systematic review registration number. | 2 – 3 |
| **INTRODUCTION** | | |  |
| Rationale | 3 | Describe the rationale for the review in the context of what is already known. | 2 |
| Objectives | 4 | Provide an explicit statement of questions being addressed with reference to participants, interventions, comparisons, outcomes, and study design (PICOS). | 2 |
| **METHODS** | | |  |
| Protocol and registration | 5 | Indicate if a review protocol exists, if and where it can be accessed (e.g., Web address), and, if available, provide registration information including registration number. | 3 |
| Eligibility criteria | 6 | Specify study characteristics (e.g., PICOS, length of follow-up) and report characteristics (e.g., years considered, language, publication status) used as criteria for eligibility, giving rationale. | 6 – 7 |
| Information sources | 7 | Describe all information sources (e.g., databases with dates of coverage, contact with study authors to identify additional studies) in the search and date last searched. | 6 – 7 |
| Search | 8 | Present full electronic search strategy for at least one database, including any limits used, such that it could be repeated. | 6 |
| Study selection | 9 | State the process for selecting studies (i.e., screening, eligibility, included in systematic review, and, if applicable, included in the meta-analysis). | 6 – 7 |
| Data collection process | 10 | Describe method of data extraction from reports (e.g., piloted forms, independently, in duplicate) and any processes for obtaining and confirming data from investigators. | 7 – 9 |
| Data items | 11 | List and define all variables for which data were sought (e.g., PICOS, funding sources) and any assumptions and simplifications made. | 7 – 9 |
| Risk of bias in individual studies | 12 | Describe methods used for assessing risk of bias of individual studies (including specification of whether this was done at the study or outcome level), and how this information is to be used in any data synthesis. | 9 |
| Summary measures | 13 | State the principal summary measures (e.g., risk ratio, difference in means). | 8 – 9 |
| Synthesis of results | 14 | Describe the methods of handling data and combining results of studies, if done, including measures of consistency (e.g., I^2^) for each meta-analysis. | 8 – 9 |

*From:*  Moher D, Liberati A, Tetzlaff J, Altman DG, The PRISMA Group (2009). Preferred Reporting Items for Systematic Reviews and Meta-Analyses: The PRISMA Statement. PLoS Med 6(7): e1000097. doi:10.1371/journal.pmed1000097

| **Section/topic** | **#** | **Checklist item** | **Reported on page #** |
| --- | --- | --- | --- |
| Risk of bias across studies | 15 | Specify any assessment of risk of bias that may affect the cumulative evidence (e.g., publication bias, selective reporting within studies). | 8 – 9 |
| Additional analyses | 16 | Describe methods of additional analyses (e.g., sensitivity or subgroup analyses, meta-regression), if done, indicating which were pre-specified. | 8 – 9 |
| **RESULTS** | | |  |
| Study selection | 17 | Give numbers of studies screened, assessed for eligibility, and included in the review, with reasons for exclusions at each stage, ideally with a flow diagram. | 10 |
| Study characteristics | 18 | For each study, present characteristics for which data were extracted (e.g., study size, PICOS, follow-up period) and provide the citations. | 11 |
| Risk of bias within studies | 19 | Present data on risk of bias of each study and, if available, any outcome level assessment (see item 12). | 12 – 13 |
| Results of individual studies | 20 | For all outcomes considered (benefits or harms), present, for each study: (a) simple summary data for each intervention group (b) effect estimates and confidence intervals, ideally with a forest plot. | 11 – 12 |
| Synthesis of results | 21 | Present results of each meta-analysis done, including confidence intervals and measures of consistency. | 11 – 12 |
| Risk of bias across studies | 22 | Present results of any assessment of risk of bias across studies (see Item 15). | 13 – 14 |
| Additional analysis | 23 | Give results of additional analyses, if done (e.g., sensitivity or subgroup analyses, meta-regression [see Item 16]). | 11 – 12 |
| **DISCUSSION** | | |  |
| Summary of evidence | 24 | Summarize the main findings including the strength of evidence for each main outcome; consider their relevance to key groups (e.g., healthcare providers, users, and policy makers). | 14 – 16 |
| Limitations | 25 | Discuss limitations at study and outcome level (e.g., risk of bias), and at review-level (e.g., incomplete retrieval of identified research, reporting bias). | 16 – 17 |
| Conclusions | 26 | Provide a general interpretation of the results in the context of other evidence, and implications for future research. | 17 |
| **FUNDING** | | |  |
| Funding | 27 | Describe sources of funding for the systematic review and other support (e.g., supply of data); role of funders for the systematic review. | 17 - 18 |

*From:*  Moher D, Liberati A, Tetzlaff J, Altman DG, The PRISMA Group (2009). Preferred Reporting Items for Systematic Reviews and Meta-Analyses: The PRISMA Statement. PLoS Med 6(7): e1000097. doi:10.1371/journal.pmed1000097

**Appendix 2. Literature search strategy**

**Early Nasogastric Tube Removal:**

Database: Embase Classic+Embase <1947 to 2019 June 12>, Ovid MEDLINE(R) ALL <1946 to June 12, 2019>

Search Strategy:

--------------------------------------------------------------------------------

1 esophagus resection/ (20422)

2 ((oesophag* or esophag*) adj2 resect*).tw. (9562)

3 (Esophagectom* or oesophagectom*).tw. (25329)

4 1 or 2 or 3 (37367)

5 exp nasogastric tube/ (10194)

6 ((nasogastric or naso gastric) adj (tub* or intub*)).tw. (13114)

7 *digestive tract intubation/ (1661)

8 (naso gastric or nasogastric).ti. (4030)

9 5 or 6 or 7 or 8 (20907)

10 4 and 9 (345)

11 decompression.mp. (109331)

12 decompress*.tw. (100869)

13 11 or 12 (121646)

14 10 and 13 (58)

15 (exp animal/ or nonhuman/ or animal experiment/) not exp human/ (11589225)

16 conference abstract.pt. (3431216)

17 14 not (15 or 16) (48)

18 limit 17 to english language (43)

19 18 use emczd (29)

20 Esophagectomy/ (28384)

21 (esophagectom* or oesophagectom*).tw,kw. (25817)

22 ((esophag* or oesophag*) adj2 resect*).tw. (9562)

23 20 or 21 or 22 (39930)

24 ((nasogastric or naso gastric) adj (tub* or intub*)).tw. (13114)

25 nasogastric.ti,kf. (4021)

26 *Intubation, Gastrointestinal/ (6023)

27 naso gastric.ti. (114)

28 or/24-27 (18305)

29 23 and 28 (274)

30 DECOMPRESSION, SURGICAL/ (31668)

31 decompress*.tw,kf. (101460)

32 30 or 31 (111436)

33 29 and 32 (52)

34 exp animals/ not humans/ (18301193)

35 33 not 34 (34)

36 limit 35 to english language (31)

37 36 use medall (20)

38 19 or 37 (49)

39 remove duplicates from 38 (32)

40 39 use medall (20)

41 39 use emczd (12)

**Early Oral Intake:**

Database: Embase Classic+Embase <1947 to 2019 June 12>, Ovid MEDLINE(R) ALL <1946 to June 12, 2019>

Search Strategy:

--------------------------------------------------------------------------------

1 Esophagectomy/ (28384)

2 (esophagectom* or oesophagectom*).tw,kw. (25817)

3 ((esophag* or oesophag*) adj2 resect*).tw. (9562)

4 or/1-3 (39930)

5 Enteral Nutrition/ and (oral*.tw. or exp eating/) (5955)

6 ((oral or liquid) and (nutrition or food or feeding* or diet*)).ti. (11779)

7 ((oral or liquid) and (nutrition or food or feeding* or diet*)).kf. (1709)

8 (early adj3 (nutrition or food or feeding* or diet*)).tw. (20042)

9 (("day 1" or 1 day) adj3 (nutrition or feeding or diet*)).tw. (1242)

10 or/5-9 (38862)

11 4 and 10 (347)

12 limit 11 to english language (303)

13 12 use medall (101)

14 esophagus resection/ (20422)

15 ((oesophag* or esophag*) adj2 resect*).tw. (9562)

16 (Esophagectom* or oesophagectom*).tw. (25329)

17 14 or 15 or 16 (37367)

18 (enteric feeding/ or feeding/) and oral*.tw. (12079)

19 (early adj3 (nutrition or food or feeding* or diet*)).tw. (20042)

20 (("day 1" or 1 day) adj3 (nutrition or feeding or diet*)).tw. (1242)

21 or/18-20 (32669)

22 17 and 21 (347)

23 limit 22 to english language (314)

24 23 use emczd (258)

25 13 or 24 (359)

**26 remove duplicates from 25 (278)**

**27 26 use medall (101)**

**28 26 use emczd (177)**

**Appendix 3. A revised tool to assess risk of bias in randomized trials (RoB 2)^1^**

| **Bias** | **Random sequence generation (selection bias)** | **Allocation concealment (selection bias)** | **Blinding of participants and researchers (performance bias)** | **Blinding of outcome assessment (detection bias)** | **Incomplete outcome data (attrition bias)** | **Selective reporting (reporting bias)** | **Publication bias** |  |
| --- | --- | --- | --- | --- | --- | --- | --- | --- |
| **EOI studies** | | | | | | | | |
| Mahmoodzadeh 2015 | Low | Low | Low | Low | Low | Low | Low |  |
| Sun 2018 | Some concerns (randomization generation methods not reported) | HIGH (no allocation concealment) | HIGH (Authors reported no blinding was used) | HIGH (Authors reported no blinding was used) | Low | Low | Low |  |
| **Early NG removal studies** | | | | | | | | |
| Daryaei 2009 | Low | Low | Low | Low | Low | Low | Low |  |
| Hayashi 2019 | Some concerns (randomization reported, procedure not reported) | Some concerns no allocation concealment reported | HIGH (Authors did not report whether blinding was used) | Some concerns, not reported (but AL is an objective measure so detection bias would likely be minimal) | Low | Low | Low |  |
| Mistry 2012 | Low | Some concerns no allocation concealment reported | HIGH (Authors did not report whether blinding was used) | Some concerns, not reported (but AL is an objective measure so detection bias would likely be minimal) | Low | Low | Low |  |
| Shackcloth 2006 | Low | Some concerns no allocation concealment reported | Some concerns, unclear whether all investigators were blinded | Low | Low | Low | Low |  |

^1^Risk of Bias: Green, low; yellow, some concerns; red, high.

**Appendix 4. Grading of Recommendations, Assessment, Development and Evaluations for Anastomotic Leak**

| **Intervention** | **Limitations in study designs or execution (ROB)** | **Imprecision** | **Inconsistency** | **Indirectness** | **Publication bias** | **Factors that increase quality of effect^1^** | **Quality of Evidence** (graded out of 4 levels^*^) |
| --- | --- | --- | --- | --- | --- | --- | --- |
| EOI (2 studies) | High risk (-2 levels) | High (-1 level)  *95% CI included the null effect* | High (-1 level) Due to moderate heterogeneity | Low | Low risk | None | **Very low quality** |
| Early NGT removal (3 studies) | Unclear risk (-1 levels) | High (-1 level)  *95% CI included the null effect* | Low | Low | Low risk | None | **Low quality** |

| GRADE: Working group grades of evidence  **High quality:** More research very unlikely to change the estimate of effect  **Moderate quality:** Means further research is likely to have an important impact on our confidence in the estimate of effect and may alter the estimate  **Low quality:** means that the effect estimate is limited and may substantially differ from  **Very low quality:** grade means that we have little confidence in the effect estimate |
| --- |

^1^Large magnitude of effect (increase 1 or 2 levels); All plausible confounding would reduce the demonstrated effect or increase the effect if no effect was observed (increase 1 level); Dose-response gradient (increase 1 level)

^2^Optimal information size (OIS) calculated using the pooled incidence rates for patients who received the stapled anastomosis intervention and patients who received the hand-sewn anastomosis, 5% and 5.8% respectively. An α 0.05, β 0.2, and a ratio of 1:1 for the comparison groups were used. The OIS was determined to be 12529 per group (or 25058 total), which was not met.
